# Acknowledgement to Reviewers of *Antibiotics* in 2018

**DOI:** 10.3390/antibiotics8010005

**Published:** 2019-01-10

**Authors:** Antibiotics Editorial Office

**Affiliations:** MDPI, St. Alban-Anlage 66, 4052 Basel, Switzerland

Rigorous peer-review is the corner-stone of high-quality academic publishing. The editorial team greatly appreciates the reviewers who contributed their knowledge and expertise to the journal’s editorial process over the past 12 months. In 2018, a total of 108 papers were published in the journal, with a median time to first decision of 15.5 days and a median time to publication of 37 days. The editors would like to express their sincere gratitude to the following reviewers for their cooperation and dedication in 2018:
Aabenhus, RuneLewis, DavidAbboud, MartineLognay, GeorgesAbejón, RicardoLohman, JeremyAldred, KatieLouie, StaceyAndrianasolo, EricLous, JørgenArango, Rachel A.Luedtke, BrandonArmstrong, GlenLuhavaya, HannaArndt, Patrick G.Manjunath, ManuboluAsamizu, ShumpeiMarcone, Giorgia LetiziaAshiru-Oredope, DianeMarques, ClaudiaAshish, PathakMartino, SabataAvent, MinyonMathieu, JacquesBach, HoracioMatsuda, YudaiBaerga-Ortiz, AbelMendes, Marta V.Baliraine, FrederickMesquita, JoãoBardi, GiuseppeMetsä-Ketelä, MikkoBeld, JorisMillard, Andrew D.Berkes, CharlotteMinami, MasaakiBernardini, GiuliaMiotla, PawelBøgwald, JarlMoll, Gert N.Briani, FedericaMotwani, TinaBrown, StanleyMurray, Thomas ScotBugla-Płoskońska, GabrielaNanjappa, DeepakCaffrey, PatrickNieto, Teresa PérezCain, RickyNimmagadda, AlekhyaCampos Da Rosa, Joaquim M.Nori, PriyaChamakura, KarthikNovick, Richard P.Charvat, Robert A.Nyerges, GyorgyiChattoraj, DhrubaOgawara, HiroshiChen, LongOkafor, FlorenceChiarelli, LaurentOlmos, DaniaChodosh, JamesOppong, RaymondChopra, TeenaPajunen, MariaChoudhary, Kumari SonalPapazafiropoulou, AthanasiaColeman, AnthonyPapp, JohnConstance, Jonathan E.Park, Se ChangCórdoba, GloriaPaskaleva, ElenaCriss, Alison K.Pedulla, MarisaCrossley, AlisonPeng, TengCrumlish, MagsPettus, ThomasCryle, MaxPhuoc, Nghia TruongCurrie, KayPiano, MartinaD’Andrea, Marco MariaPinto, TatianaDallinger, ReinhardPomposelli, JamesDanziger, Larry H.Puiggalí, JordiDavid, Michael Z.Pyatenko, Alexanderde la Salud-Bea, RobertoRagusa, AndreaDe Peralta, Tracy L.Rahme, KamilDe Piccoli, GiacomoRaposio, EdoardoDewulf, JeroenReid, Christopher W.Díaz Martínez, MargaritaRoh, ChanghyunDixon, NicholasRoque, FátimaDkhar, Hedwin KitdorlangSabatier, Jean-MarcDomingo-Calap, PilarSacco, Keith A.Drlica, KarlSaiz Jimenez, CesareoEckroat, ToddSantander, JavierEdwards, Adrianne N.Schaaper, RoelEl Badawy, AmroSchäberle, Till F.Else, Terry AnnSchmidt, MartinEl-Seedi, Hesham R.Schwan, WilliamEsteve-Romero, JosepSclavi, BiancaEustaquio, Alessandra S.Scrano, LauraFernández, LucíaSeekatz, Anna MariaFernandez-Martinez, LorenaSeipke, RyanFlemetakis, EmmanouilSeo, Keun-SeokFleming-Dutra, Katherine E.Setzer, William N.Floresta, GiuseppeSharkey, LiamFouladkhah, AliyarSheikh, AlaullahFranco, Carlos M.Shen, YangFurneri, Pio MariaSilvestre, SamuelGao, ZheSim, EdithGarcia, Coralia V.Skandalis, NikolaosGhosh, SantoshSmani, YounesGiacobbe, Daniele RobertoSnyder, LoriGile, GillianSorensen, JohnGoh, ShanSorg, OlivierGökce, BilalStein, DanielGomes, Maria S.Straight, PaulGoyer, ClaudiaStreubel, RobertHadjikakou, SotirisSummers, James KevinHan, QifengSutton, ScottHansford, KarlSzewczyk, KatarzynaHardies, Stephen C.Taheri, ShimaHashimoto, MasaruTailhades, JulienHaslinger, KristinaTakahashi, SatoshiHassan, AmanyTamulevičienė, AstaHayes, Sidney J.Tavío, María M.Hearn, Michael J.Tayebati, Seyed KhosrowHendrickson, HeatherToiu, AncaHernalsteens, Jean-PierreTonkin-Crine, SarahHindra, HindraTorres-Barceló, ClaraHirakawa, HidetadaToscani, AnitaHolmes, Neil A.Tyndall, JoelHuang, Cheng-LiangUeda, KenjiIto, TakuyaValencia, GregorioJain, PrashantValera, EnriqueJankauskaite, VirginijaVamanu, EmanuelJay-Russell, MicheleVan Boeckel, ThomasJohnson, PaulVan Lanen, StevenJones, MarjorieVasile, FrancescaJordao, LuisaVemula, HarikaJu, Kou-SanVenturini, CarolaKahler, CharleneViale, PierluigiKakkar, AshokVijgenboom, ErikKako, TetsuyaWagemans, JeroenKalatzis, Panos G.Wanat, MartaKappell, AnthonyWang, Liang-ChunKeck, JamesWeber, J. MarkKiljunen, SaijaWeber, TilmannKinaciyan, TamarWendling, Carolin C.Kobayashi, HidekiWhite, Kimberly N.Komaki, HisayukiWink, JoachimKondo, TatsuyaWitte, AngelaKonstantinidis, Theocharis G.Wu, ChangshengKoutelidakis, Antonios E.Xie, RanKoyama, YuYang, LeiKu, SeockmoYim, GraceKuper, KristiYu, AiminKvítek, LiborZabetakis, IoannisLaanto, ElinaZhang, FanLarabee, Jason L.Zhu, JingenLecky, Donna M.Ziemert, NadineLeung, Chung Yin (Joey)


